# Cross-sectional study of factors affecting the receipt of mental health education in older migrants in China

**DOI:** 10.1186/s12889-023-15287-6

**Published:** 2023-02-22

**Authors:** Wanchen Wang, Jia Song, Chengxin Fan, Qiusha Li, Dongping Ma, Wenqiang Yin

**Affiliations:** 1 School of Public Health, Weifang Medical University, NO.7166, Baotong West Street, Weifang, Shandong China; 2 School of Management, Weifang Medical University, NO.7166, Baotong West Street, Weifang, Shandong China

**Keywords:** Mental health education, Older migrant population, Influencing factors

## Abstract

**Background:**

Population migration in China has increasingly included middle-aged and older populations. Relatedly, the lack of mental health education among China’s older migrants is still an important but neglected problem. This study aimed to understand the current situation of mental health education received by the older migrant population in China and to explore related influencing factors.

**Methods:**

This cross-sectional study included 5729 older migrants who participated in the 2017 China Migrants Dynamic Survey. The independent variables included four components: demographic and socioeconomic characteristics, migration factors, public health service utilization, and social integration factors. Data were analyzed using the chi-square test for single factors and a binary logistic regression model for multiple factors.

**Results:**

A total of 1749 older migrants received mental health education, for a receipt rate of 30.53%. Regression analysis showed that older migrant individuals who had an average monthly household income > 3000 CNY, self-rated their health as healthy, had chronic diseases, had heard of National Basic Public Health Services, had established health records, received ≥2 types of health education were willing to integrate into the local population, and were highly involved in the community were more likely to receive mental health education. Older migrants who were ≥ 70 years old, had an elementary school education or below, had difficulties in the local community, had migrated ≥11 years prior, moved for their offspring, and moved across provinces were less likely to receive mental health education.

**Conclusions:**

The older migrant population does not receive sufficient mental health education. Mental health interventions should be tailored to the characteristics of older migrants to increase their mental health literacy and meet their psychological needs.

## Background

Migration is an important event in individuals’ lives and significantly affects their health. The International Organization of Migration estimates that by 2020, 281 million people, or 3.6% of the global population, will have migrated internationally [1]. Chinese internal migration is one of the largest migration flows in the world [2]. The number of people living in places other than their household registration areas reached approximately 490 million, and the migrant population reached approximately 375 million, according to the latest statistics from China’s National Bureau of Statistics [3]. The size of the migrant population is changing and is showing new features, with an increasing number of middle-aged and older people and an increasing proportion of older migrants in terms of age structure and size [4]. In addition to the individual’s quality of life, family happiness, and macroeconomic development, the health of the older migrant population is also an ongoing issue of concern for scholars worldwide [5–7].

In terms of mental health problems, older migrants, unlike younger migrants, are more susceptible to the negative effects of migration factors on their mental well-being [8]. Several cross-sectional studies have reported a high prevalence of mental health problems among older migrant populations; for example, there has been research on depression among Asian immigrants of Chinese and Korean descent living in the United States [9, 10] and immigrants of Mexican descent [11], and another study with older immigrants in 11 European countries also reported a higher prevalence of depression among immigrants aged 50 and above compared to residents with no migration history [12]. Various studies have shown that psychological problems in older migrant populations are caused mainly by factors other than diseases, such as identity, income, cultural environment, and societal support [13–16]. For instance, older adults who move to a new country with their children to attend school or work may find it difficult to change their original language, behavior, and lifestyle to adapt to the changing environment, and they may become less integrated into the local community. Therefore, the significant changes in social life due to migration have a specific psychological impact on older migrants, and the effects of various social factors increase the mental health risk for the older migrant population.

The older migrant population faces more mental health risks and has lower mental health due to both their migration and old age. Their demand for health education is relatively high, as they require more effective interventions to enhance their health awareness and mental health literacy [17]. Mental health education helps older people understand basic knowledge about psychological cognition and basic skills and methods to address psychological problems. It also helps reduce negative emotions such as anxiety and depression, which helps to improve older people’s physical and mental health [18].

Therefore, to understand the receipt of mental health education among the older migrant population and the influencing factors, this study uses data from the 2017 China Migrants Dynamic Survey (CMDs). The objective of this study was to assess the main factors affecting the receipt of mental health education among older migrants and their migration characteristics. Furthermore, we provide a reference point for promoting and improving mental health education and health literacy policy formulation and practice among older migrants.

## Methods

### Data collection and participants

**Fig. 1:**
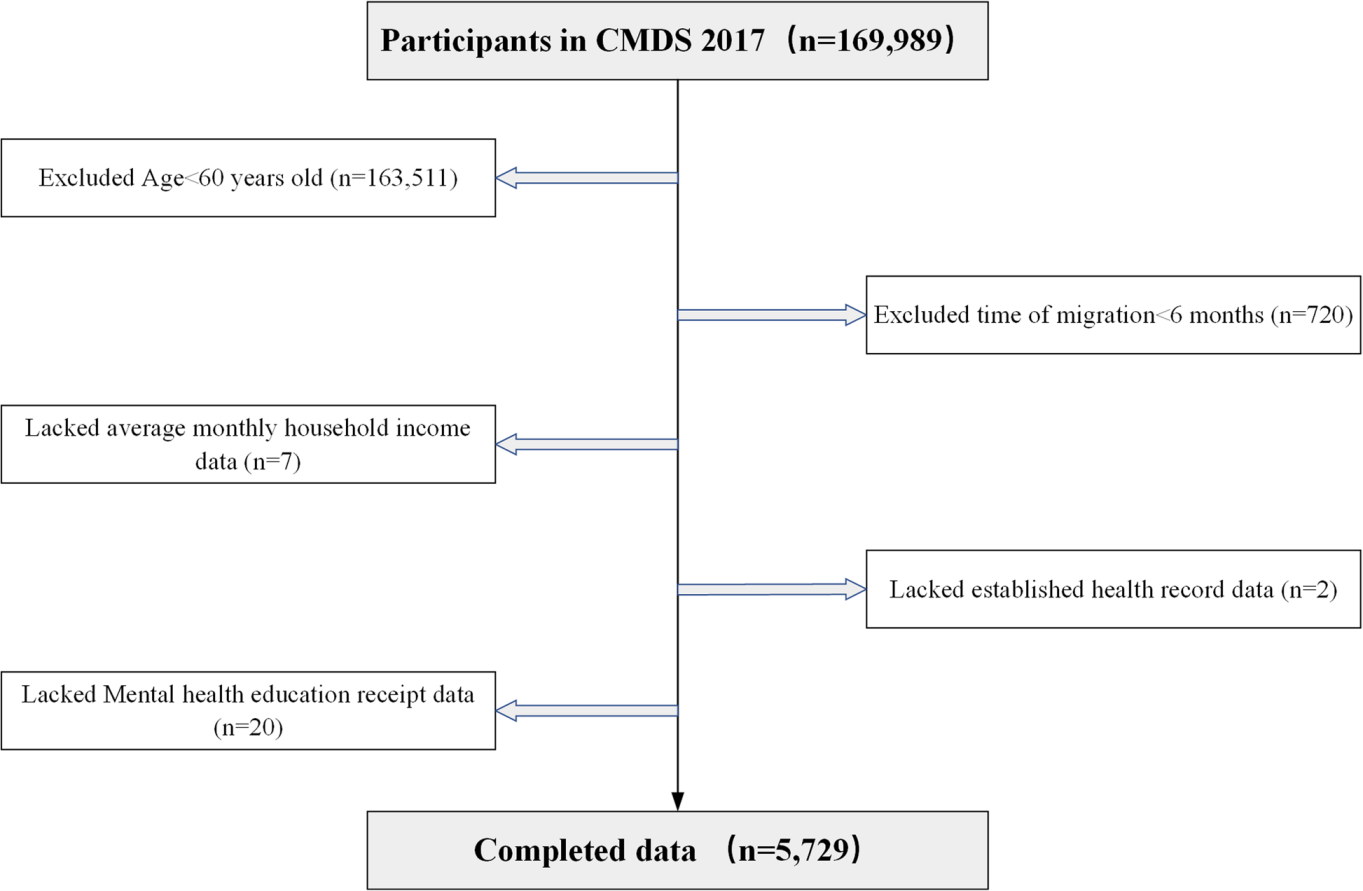
Sample selection flowchart

The 2017 CMDs was conducted in May 2017 in inflow areas where the migrant population is more concentrated in a total of 31 provinces (autonomous regions and municipalities) and the Xinjiang Production and Construction Corps in China. Individuals who were residing in the local area for ≥1 month, had nonlocal residence, and were aged ≥15 years old in May 2017 were selected as the survey population. Dynamic monitoring of the migrant population was conducted using a stratified, multistage, and proportional sampling method. Migrants refer to persons whose residence does not coincide with the place of household registration and who have been away for at least 6 months [19]. Moreover, the migrant population can receive mental health education services only if they have resided in the inflow area for ≥6 months. Therefore, participating subjects were older adults who had lived in the inflow area for ≥6 months and were ≥ 60 years old, and after samples with missing relevant variables were excluded, the final valid sample size was 5729 (Fig. 1).

### Dependent variable

The following question was used to measure the receipt of mental health education: “In the past year, have you received health education on mental health in your current village/residence council?” (0 = no, 1 = yes).

### Independent variables

Independent variables included gender, marital status, age, socioeconomic status (education level, average monthly household income, experience of difficulties in the local area), health status (self-rated health status, chronic diseases), migration factors (duration of migration, reasons for migration, range of migration), public health service utilization (awareness of the National Basic Public Health Service, possession of established health records, received ≥2 types of health education), and community integration factors (identification as local people, community involvement, and willingness to integrate). Studies have shown that several risk factors are associated with increased mental health risks among migrants, including personal characteristics (age, gender, education, etc.), factors related to the social environment of migration, post-migration stress factors, etc. [20, 21]. Although the risk factors involved in the process of population aging are broadly similar, migrant-specific risk factors may also play a relevant role in influencing the health and quality of life of older adults and include the reason for migration, the extent of migration, and the timing of migration [22]. Non-communicable diseases (NCDs) and self-rated health status are often reported as risk factors for deterioration in mental health [23, 24]. There is a clear association between lower socioeconomic status and poorer mental health status [25]. Greater wealth is often associated with better access to health care, leading to better disease management and better health status. In China, the hukou system determines people’s eligibility for a range of socioeconomic benefits provided by the state [26]. Older migrants, as a non-household resident population, has difficulty in enjoying the same public services such as healthy aging, health care, and social services as the household population. Whereas social support and a strong network of basic public health services can buffer the stressful effects of the migration process and influence mental health, the use of basic public health services has an impact on the physical and mental health of migrants [27]. As an important part of the migrant population, the older migrant population is a relatively vulnerable group in the migrant population, and the willingness of this migrant population to integrate in their new place of residence, the objective degree of social integration, and the determination and satisfaction of their own social integration are all related to their physical and mental health, so social integration factors have an important influence on the mental health of the older migrant population [28, 29].

Among these variables, experience of difficulties in the local community was assessed with the following question: “Do you have any difficulties in the local community at present?” Willingness to integrate was measured by the item, “I am willing to integrate with the local people and become one of them.” Community involvement was based on the questions, “Have you made suggestions to your unit/community/village or monitored the management of unit/community/village affairs since 2016?” and “Have you participated in charitable efforts, blood donations, volunteer activities since 2016?” (1 = no, 2 = occasionally, 3 = sometimes, 4 = often). The scores of these two questions were summed, and the total scores obtained for community involvement were divided into three categories with reference to Yang’s study [30]: 2 was classified as low community involvement, 3 ~ 4 = moderate community involvement, and 5 ~ 8 = high community involvement.

### Statistical analysis

The data were analyzed using SPSS version 25.0 (SPSS, Chicago, Illinois, USA). We present descriptive statistics. The results are expressed as the number (proportion) for the categorical variables of the older migrant population. The chi-square test and binary logistic regression analysis were used to investigate factors affecting the receipt of mental health education among older migrants.

## Results

### Participants’ demographic characteristics

Table 1 presents the sociodemographic information of the 5729 survey respondents. Among them, 1749 older migrant people received mental health education, for a receipt rate of 30.53%. More than half were male (3303, 57.65%), the average age was 66.39 ± 5.55 years, most were in the age range of 60–69 years (4480, 78.20%), and most were married (4789, 83.59%). The educational level was low, and almost half had a primary school education or below (2767, 48.30%). The rest of the details can be viewed in Table 1.

**Table 1:** Descriptive statistics of the study sample

Variable		Measurement	n (%)/mean (SD)
Sociodemographic characteristics	Gender	Female = 0	2426 (42.35)
		Male = 1	3303 (57.65)
	Age (years)	60 ~ 69 = 1	4480 (78.20)
		70 ~ 79 = 2	1077 (18.80)
		≥80 = 3	172 (3.00)
	Marital status	Married = 0	4789 (83.59)
		Other = 1	940 (16.41)
	Education	Primary School or below = 1	2767 (48.30)
		Junior High School = 2	1716 (29.95)
		High school = 3	875 (15.27)
		College and above = 4	371 (6.48)
	Average monthly household income (yuan)	≤CNY 3000 = 1	1833 (32.00)
		CNY 3001 ~ 6000 = 2	2185 (38.14)
		≥CNY 6001 = 3	1711 (29.87)
	Difficulties in the local community	No = 0	2657 (46.38)
		Yes = 1	3072 (53.62)
	Self-rated health	Unhealthy = 0	1110 (19.38)
		Healthy = 1	4619 (80.62)
	Chronic diseases	No = 0	3656 (63.82)
		Yes = 1	2073 (36.18)
Factors of population migration	Years since migration (years)	≤5 = 1	2495 (43.55)
		6 ~ 10 = 2	1481 (25.85)
		≥11 = 3	1753 (30.60)
	Migrating for offspring	No = 0	3360 (58.65)
		Yes = 1	2369 (41.35)
	Migration range	Intercounty within the city = 1	1211 (21.14)
		Intercity in the province = 2	1998 (34.88)
		Interprovincial =3	2520 (43.99)
Utilization of public services	Awareness of National Basic Public Health Services	No = 0	2461 (42.96)
		Yes = 1	3268 (57.04)
	Established health record	No = 0	3837 (66.98)
		Yes = 1	1892 (33.02)
	Received ≥2 types of health education	No = 0	2949 (51.47)
		Yes = 1	2780 (48.53)
Social integration factors	Identifies as a local	No = 0	649 (11.33)
		Yes = 1	5080 (88.67)
	Involvement in community activities	Insufficient involvement = 1	4300 (75.06)
		Moderate involvement = 2	1121 (19.57)
		High involvement = 3	308 (5.38)
	Willingness to integrate	Disagree = 1	249 (4.35)
		Basically agree = 2	2369 (41.35)
		Totally agree = 3	3111 (54.30)

### Mental health education currently received by the older migrant population

The results of the chi-square test are presented in Table 2. Individuals with different characteristics in the older migrant population responded differently regarding mental health education. Specifically, the differences in the receipt of mental health education among older migrants based on the 15 independent variables were statistically significant (*P* < 0.05), except for those based on gender and marital status, which were not statistically significant (*P* > 0.05).

**Table 2:** Participants’ demographic characteristics

Variable	Subgroups	Mental health education receipt	χ2	***p value***s
		n (%)/mean (SD)		
Gender	Female	711 (29.31)	0.992	0.319
	Male	1038 (31.43)		
Age (years)	60 ~ 69	1390 (31.03)	5.973	0.015
	70 ~ 79	321 (29.81)		
	≥80	38 (22.09)		
Marital status	Married	1487 (31.05)	0.069	0.792
	Other	262 (27.87)		
Education	Primary school or below	721 (26.06)	25.827	< 0.001
	Junior high school	562 (32.75)		
	High school	321 (36.96)		
	College and above	145 (39.08)		
Average monthly household income (yuan)	≤CNY 3000	451 (24.60)	23.097	< 0.001
	CNY 3001 ~ 6000	784 (35.88)		
	≥CNY 6001	514 (30.04)		
Difficulties in the local community	No	1011 (38.05)	10.890	0.001
	Yes	738 (24.02)		
Self-rated health	Unhealthy	249 (22.43)	15.799	< 0.001
	Healthy	1500 (32.47)		
Chronic diseases	No	1104 (30.20)	8.760	0.003
	Yes	645 (31.11)		
Years since migration (years)	≤5	720 (28.86)	9.757	0.016
	6 ~ 10	500 (33.76)		
	≥11	529 (30.18)		
Migrated for offspring	No	1138 (33.87)	20.195	< 0.001
	Yes	611 (25.79)		
Migration range	Intercounty within the city	388 (32.04)	17.254	< 0.001
	Intercity in the province	652 (32.63)		
	Interprovincial	709 (28.13)		
Awareness of National Basic Public Health Services	No	362 (14.71)	476.763	< 0.001
	Yes	1387 (42.44)		
Established health record	No	889 (23.17)	297.451	< 0.001
	Yes	860 (45.45)		
Received ≥2 types of health education	No	761 (25.81)	46.515	< 0.001
	Yes	988 (35.54)		
Identifies as a local	No	147 (22.65)	21.421	< 0.001
	Yes	1602 (31.54)		
Involvement in community activities	Insufficient involvement	1053 (24.49)	215.399	< 0.001
	Moderate involvement	517 (46.12)		
	High involvement = 3	179 (58.12)		
Willingness to integrate	Disagree = 1	56 (22.40)	8.145	0.004
	Basically agree = 2	618 (25.60)		
	Totally agree = 3	1075 (35.07)		

### Analysis of the factors influencing the receipt of mental health education among the older migrant population

A binary logistic regression analysis was conducted to determine whether the older migrant population had received health education on mental health in the past year as the dependent variable (0 = no, 1 = yes) and statistically significant independent variables. The results, shown in Table 3, indicated that older migrant adults who had a junior high school education and above, had an average monthly household income > 3000 CNY, self-rated their health as healthy, suffered from chronic diseases, had heard about National Basic Public Health Services, had established local health records, received ≥2 types of health education, identified as a local, had high levels of community involvement, and were fully willing to integrate into the local population were more likely to receive mental health education. Those who were ≥ 70 years old, had difficulties in the local community, had migrated ≥11 years prior, migrated for offspring, and moved across provinces did not tend to obtain mental health education.

**Table 3:** Analysis of the factors influencing the receipt of mental health education among older Chinese migrants

Variable	Subgroups	β	SE	Wald ***χ2***	***p value***s	Odds Ratio	95% CI
Age (years)	60 ~ 69	–	–	–	–	–	–
	70 ~ 79	−0.258	0.081	3.518	0.012	0.244	0.205 ~ 0.715
	≥80	−0.407	0.200	4.134	0.023	0.665	0.449 ~ 0.985
Education	Primary school or below	–	–	–	–	–	–
	Junior high school	0.332	0.274	12.455	0.006	1.551	1.210 ~ 1.914
	High school	0.871	0.394	14.972	< 0.001	2.135	1.951 ~ 3.355
	College and above	0.921	0.551	17.003	< 0.001	2.288	2.005 ~ 2.649
Average monthly household income (yuan)	≤CNY 3000	–	–	–	–	–	–
	CNY 3001 ~ 6000	0.432	0.518	4.160	0.007	1.302	1.188 ~ 1.713
	≥CNY 6001	0.574	0.788	4.394	< 0.001	1.688	1.459 ~ 1.822
Difficulties in the local area	No = 0	–	–	–	–	–	–
	Yes = 1	−0.145	0.064	4.960	< 0.001	0.456	0.143 ~ 0.691
Self-rated health	Unhealthy	–	–	–	–	–	–
	Healthy	0.257	0.488	8.534	0.003	1.292	1.088 ~ 1.535
Chronic diseases	No	–	–	–	–	–	–
	Yes	0.803	0.036	9.416	< 0.001	1.448	1.164 ~ 1.867
Years since migration (years)	≤5	–	–	–	–	–	–
	6 ~ 10	0.002	0.075	0.537	0.983	1.002	0.865 ~ 1.160
	≥11	−0.156	0.174	4.383	0.006	0.856	0.740 ~ 0.990
Migrated for offspring	No	–	–	–	–	–	–
	Yes	− 0.332	0.068	24.016	< 0.001	0.717	0.628 ~ 0.819
Migration range	Intercounty within the city	–	–	–	–	–	–
	Intercity in the province	0.048	0.084	0.332	0.565	1.049	0.891 ~ 1.236
	Interprovincial	−0.391	0.184	4.182	0.001	0.613	0.577 ~ 0.976
Awareness of National Basic Public Health Services	No	–	–	–	–	–	–
	Yes	1.115	0.075	220.106	< 0.001	3.050	2.633 ~ 3.535
Established health record	No	–	–	–	–	–	–
	Yes	0.501	0.069	52.341	< 0.001	1.651	1.441 ~ 1.891
Received ≥2 types of health education	No	–	–	–	–	–	–
	Yes	0.318	0.032	93.761	< 0.001	1.371	1.113 ~ 1.697
Identifies as a local	No	–	–	–	–	–	–
	Yes	0.178	0.111	2.587	0.108	1.195	0.962 ~ 1.484
Involvement in community activities	Insufficient involvement	–	–	–	–	–	–
	Moderate involvement	0.339	0.072	22.263	< 0.001	1.404	1.219 ~ 1.617
	High involvement	0.426	0.041	107.866	< 0.001	1.531	1.413 ~ 1.660
Willingness to integrate into the local population	Disagree	–	–	–	–	–	–
	Basically agree	0.062	0.173	0.126	0.722	1.063	0.758 ~ 1.493
	Totally agree	1.383	0.078	4.899	< 0.001	1.467	1.045 ~ 2.060

## Discussion

The migration and instability of the older migrant population make their mental health easy to neglect [31]. Mental health education helps aging individuals understand basic knowledge about their psychological cognition and develop basic skills and methods to address psychological problems, prevent negative attitudes and reduce negative emotions such as anxiety and depression, which helps to enhance their physical and mental health [32–35]. Generally, the results of this study demonstrated that the average receipt rate of mental health education among the older migrant population aged ≥60 years in China was 30.53%. There is a considerable gap in the goal of “by 2020, the health education coverage rate of the migrant population will be>95%” in the 13th Five-Year National Health and Family Planning Service Management Plan for the migrant population. The mental health education of older migrants still needs to be improved [36].

The age, socioeconomic status, self-rated health status, chronic disease status, and migration characteristics of older migrants significantly influenced their receipt of mental health education services. The lower receipt rate of mental health education among those aged ≥70 years old may be related to the fact that older migrants have fewer visible mental health problems than physical illnesses and, therefore, pay more attention to their physical health status and neglect their mental health status [37]. The receipt of mental health education among the older population increased with the education level, which may be related to the fact that people with higher education levels pay more attention to their psychological problems and acquire mental health knowledge [38]. There was lower receipt of mental health education among older migrants with local difficulties, which may be because these migrants have no time to pay attention to mental health education and weaker access to it because of their local life pressures or other challenges [39]. In addition, the higher receipt rate of mental health education among older migrants who self-rated their health as healthy suggests that health education can be translated into health literacy and health awareness, which can improve health status [40]. Migrant older adults with chronic diseases were more eager to obtain medical advice to improve their awareness of diseases as well as their psychological situations [41, 42]. Currently, the household registration system still affects residents’ utilization of public health services, the accessibility of health services for the migrant population is low [43, 44], and the acceptance rate of mental health education among older adults who move across provinces is low due to a lack of knowledge about local health services. The reception rate of mental health education was lower among older individuals with ≥11 years since migration. In the early stages of migration, the older migrants have a lower level of social adjustment and the stress of the external environment can have a greater impact on their psychological well-being. Zhang confirmed in his study that the length of time since migration positively predicted positive mood [45]. Liu also found that older migrants who had migrated more than 10 years prior reported better subjective well-being than they had experienced in their place of origin [46], and over time, the unfamiliar environment’s barriers to adaptation gradually decrease, the level of social integration continued to increase, and their mental health improved [47]. Thus, the need for mental health education decreased. The reception rate of mental health education among older migrants for their offspring is low. Many older people have to be forced to take on the responsibility of caring for their minor grandchildren in the face of the absence of family care for their children and social childcare services are still inadequate [48]. Therefore, they may be trapped in the caregiver role responsibilities, focus their lives too much on family life, and lose their own activity schedule without having free time to receive mental health education, making the receipt of mental health education poor [49]. Thus, the health administration should focus on migrants who are older, have low education levels, have difficulties in the local area, migrated for their offspring, and moved across provinces, and they should include them in the scope of mental health management services in the inflow area according to the system of allocating service resources to the resident population [50].

The utilization of basic public health services and community integration factors are essential facilitators of mental health education among older migrants [51–54]. This may be because health education is one of the main components of the National Basic Public Health Service Program, and older migrants who have heard of this program or received other health education are more familiar with the mental health education service participation process and policies. In addition, older migrants are more likely to be exposed to mental health education campaigns when establishing health records in primary health institutions and to have a stronger initiative to receive mental health education. Therefore, the community should increase the publicity and awareness rate of National Basic Public Health Services so that older migrants have a deeper understanding of the specific contents of the services and the ways to participate [55]. However, due to older migrants’ weak mental health awareness, they do not actively contact or obtain mental health education-related services, and it is difficult for them to obtain timely information about free health lectures and propaganda provided by relevant government departments. All regions in China are currently promoting community-based family doctor contracting services to establish long-term, stable service relationships between family doctors and residents. Because of this, health institutions can rely on community family doctors to establish mental health files and provide mental health tracking services for older migrants in their service area in the cell grid to follow the biopsychosocial medicine model and increase the content and frequency of services such as home visits, health monitoring, and intervention [56]. The application of the grid model can effectively ensure that all older people can enjoy mental health services [57]. Mental health professionals are recruited to provide face-to-face mental health services for older migrants. In addition, there was a significant positive correlation between the community involvement of older migrants and their mental health education receipt rate; the higher the participation of older migrants in community affairs was, the higher their mental health education receipt rate. It is suggested that health education-related services require the awareness and active participation of service recipients, and the increase in service supply alone does not necessarily promote an increase in the utilization rate. The better receipt of mental health education among older migrants with a strong willingness to integrate may be related to the fact that only when migrants are willing to integrate into the local population will they have a strong sense of belonging and identification with the inflow area and be more inclined to seek mental health education services [58]. Therefore, the community should encourage older migrants to integrate more actively into the inflow area and participate in community activities to ensure that they can enjoy the same quality of mental health education services as residents in the inflow area.

### Limitations

The study has several limitations that should be considered. We used data from 2017, and cross-sectional data did not allow us to determine trends or long-term associations between mental health education and related indicators among older migrant populations and could not verify causal relationships among specific mechanisms. Due to the limitations of public databases, information on each variable was only available based on the original survey questions, which prevented further exploration of other possibly relevant variables, such as life course, cross-cultural adjustment, and social relationships. In addition, mental health education receipt behavior was measured as a dichotomous variable, and this evaluation criterion was based on the premise that each province attached the same level of importance to mental health education. In fact, different provinces give different levels of attention to the health of mobile populations, which can affect their options for health education content [59]. Although the simplified criteria facilitate overall comparison, they may cause bias in the interpretation of the results. In-depth studies using health education acceptance intensity or delineating more detailed evaluation indicators could be considered in the future.

## Conclusion

With the aging population and growing migrant population, solving the mental health-related problems of the aging migrant population is a crucial component of achieving healthy and positive aging. In the future, we should conduct further in-depth studies on the utilization of mental health education services for aging migrants, develop developmental health interventions tailored to their characteristics, and comprehensively improve their health awareness and mental health literacy level through health education. Improving the mental health status of the older population, reducing the incidence of mental illness, and increasing recognition of mental illness and utilization of mental health services are fundamental to promoting healthy aging.

## Data Availability

Data can be accessed by logging into the China Migrant Population Data Platform (https://www.chinaldrk.org.cn/wjw/#/home) and following the instructions on the website to register for a free account. It is necessary to submit a research plan and an application form to the platform if you wish to obtain research data. Researchers will need to obtain authorization from their institutions prior to submitting the application form. Moreover, researchers need to sign a data use agreement with the Migrant Population Service Center of the National Health Commission, ensuring that they will use the data in accordance with the requirements of the agreement and will not disclose the data to any third parties. We provide the corresponding author’s email: yinwq1969@126.com, in case any problems are encountered by the readers, editors, and reviewers accessing the data.
